# Nuclear Integrants of Organellar DNA Contribute to Genome Structure and Evolution in Plants

**DOI:** 10.3390/ijms21030707

**Published:** 2020-01-21

**Authors:** Guo-Jun Zhang, Ran Dong, Li-Na Lan, Shu-Fen Li, Wu-Jun Gao, Hong-Xing Niu

**Affiliations:** 1 College of Life Sciences, Henan Normal University, Xinxiang 453007, China; zgj003@126.com (G.-J.Z.); randong0930@163.com (R.D.); lanlina281@163.com (L.-N.L.); lishufen83@163.com (S.-F.L.); 2 School of Basic Medical Sciences, Xinxiang Medical University, Xinxiang 453003, China

**Keywords:** genome structure, genome evolution, DNA transfer, nuclear integrants of plastid DNA (NUPT), nuclear integrants of mitochondrial DNA (NUMT)

## Abstract

The transfer of genetic material from the mitochondria and plastid to the nucleus gives rise to nuclear integrants of mitochondrial DNA (NUMTs) and nuclear integrants of plastid DNA (NUPTs). This frequently occurring DNA transfer is ongoing and has important evolutionary implications. In this review, based on previous studies and the analysis of NUMT/NUPT insertions of more than 200 sequenced plant genomes, we analyzed and summarized the general features of NUMTs/NUPTs and highlighted the genetic consequence of organellar DNA insertions. The statistics of organellar DNA integrants among various plant genomes revealed that organellar DNA-derived sequence content is positively correlated with the nuclear genome size. After integration, the nuclear organellar DNA could undergo different fates, including elimination, mutation, rearrangement, fragmentation, and proliferation. The integrated organellar DNAs play important roles in increasing genetic diversity, promoting gene and genome evolution, and are involved in sex chromosome evolution in dioecious plants. The integrating mechanisms, involving non-homologous end joining at double-strand breaks were also discussed.

## 1. Introduction

Three genetic compartments coexist in the plant cells: the nucleus and two cytoplasmic organelles, namely, chloroplast (plastid) and mitochondria. Among these cellular parts, the nucleus harbors the majority of the genetic material, and chloroplast and mitochondria contain relatively little but essential genetic substance. It is widely accepted that chloroplasts and mitochondria in eukaryotic cells are descended from erstwhile free-living organisms (α-proteobacteria and cyanobacteria) by endosymbiosis more than a billion years ago [1,2,3]. When the three genetic compartments were involved in one cell, genetic flux among them occurred frequently [4,5,6]. In theory, there are six types of DNA transfer occur among the three genetic compartments. At least five types, including mitochondrion-to-nucleus, plastid-to-nucleus, plastid-to-mitochondrion, nucleus-to-mitochondrion, and mitochondrion-to-plastid DNA transfer, have been observed presently based on experimental and bioinformatics data [4]. The transfer frequency is remarked different among the different types of genetic flow. Mitochondrion and chloroplast DNA have been integrated to the nuclear genome with high frequency, whereas other types of transfer occur rarely. In fact, along with the evolutionary process of the plastid and mitochondrion, many genes have relocated from the organelle to host nuclear genomes. Such events combined with the deletion of dispensable organelle genes considerably reduced the genome size of the mitochondrion and plastid during the evolution from their progenitors [4,7]. Organellar DNA transfer into the nuclear genome not just occurred during the establishment of symbiosis; it still actively occurs continuously [8,9,10,11,12,13]. The organelle-derived DNAs in the nucleus are designated as nuclear integrants of plastid DNA (NUPTs) and nuclear integrants of mitochondrial DNA (NUMTs). The NUPTs and NUMTs discussed here refer to the nuclear organellar DNAs, which still have homologous copies in the organelles and do not include the relocated organellar genes that were previously reviewed [4,7,14].

With the rapid development of high-throughput sequencing technology, the plastid, mitochondrial, and nuclear genomes of a number of plant species have been deciphered. This makes the large-scale analysis of NUPTs and NUMTs in plants be possible. Currently, NUPTs and NUMTs in some plant genomes have been characterized [15,16,17,18,19,20,21]. The characterization of nuclear organellar DNAs in plants adds new insights into the role of NUPTs and NUMTs in genome structure and evolution. Growing pieces of evidence has revealed that DNA transfer from organelles contributes considerably to gene and genome evolution and provides a major source of genetic diversity [4,22,23,24]. In this review, we summarized recent advances in organelle-derived sequences and genome evolution in plants, with focus on the evolutionary fate of these insertions and the roles of NUPTs/NUMTs in the structure and evolution of plant genomes.

## 2. Characterization of Organellar DNA-Derived Sequences in Plants

The nuclear integrants of organellar DNA were first discovered in a study in which a mitochondrial ATPase subunit gene was found in the nuclear genome, as well as in the mitochondrial genome of *Neurospora crassa* [25]. Since then, organelle-derived sequences were examined in the nuclear genome of a number of animals [26,27,28,29,30,31,32] and plants [15,16,17,18,19,20,21]. The availability of a large amount of plant organelle and nuclear genome data has made it possible to investigate the prevalence and characteristics of NUMTs and NUPTs in plants. In addition to some plant genomes analyzed previously, we estimated the whole genome landscape of NUMTs and NUPTs in the majority of currently sequenced plant species. A dataset of NUPTs in 199 plant genomes and NUMTs in 91 plant genomes was obtained (Tables S1 and S2). The analysis methods were described in Supplemental File 1. It should be noted that the NUPT and NUMT insertions analyzed here are all contiguous fragments reflected from the BLAST results, and the rearrangements of NUPTs/NUMTs from different regions of chloroplast/mitochondrial genomes are not analyzed.

### 2.1. Number and Size Distribution

NUPTs and NUMTs were observed in all examined plant genomes. Their number and size distributions vary markedly among different species. The average size of NUPT ranges from 57 bp (*Chlorella variabilis*) to 3382 bp (*Porphyra umbilicalis*), and the total NUPT length ranges from 1038 bp (*Phaeodactylum tricomutum*) to 9.83 Mb (*Triticum urartu*). The NUPT proportion of these genomes varies from 0.004% to more than 1% in three genomes, including those of *Cucurbita maxima*, *Porphyridium purpureum*, and *Ziziphus jujuba*. The number of NUPTs is few in the majority of algae, and the genomes with NUPT number less than 200 are all from algae. By contrast, NUPTs are abundant in most of the flowering plants, with the highest number found in *Triticum urartu* (Table S1). Similarly, NUMTs are also highly varied among different plant species. The cumulative length within the examined plant genomes varies from 327 bp in *Cyanidioschyzon merolae* to 11.42 Mb in *Capsicum annuum*, and the NUMT proportion accounting for the nuclear genome is from 0.0002% to 2.08% (Table S2). Previous studies showed that the longest NUPT is a 131-kb integrant detected in rice [33,34]; a 620-kb NUMT insertion derived from partially duplicated mitochondrial DNA investigated in *Arabidopsis* is the largest NUMT examined to date [35]. Our analysis detected a 135-kb NUPT in *Gossypium hirsutum*; thus, it is the longest NUPT insertion known so far (Table S1). The NUPT/NUMT fraction usually accounts for less than 0.1%, which is a small fraction of the nuclear genome. However, it should be noted that “old” NUPT and NUMT sequences are usually difficult to detect because of constant mutation and rearrangement during the evolutionary process [29,36]. In addition, NUMTs and NUPTs with high sequence similarity to mitochondrial/chloroplast DNA sequences may be removed as organelle contamination when the nuclear genomes are assembled. Thus, even in “thoroughly sequenced” nuclear genomes, NUMTs/NUPTs may not be completely investigated. Therefore, the content of these organelle-derived sequences is usually underestimated using the standard BLAST method.

**Figure 1:**
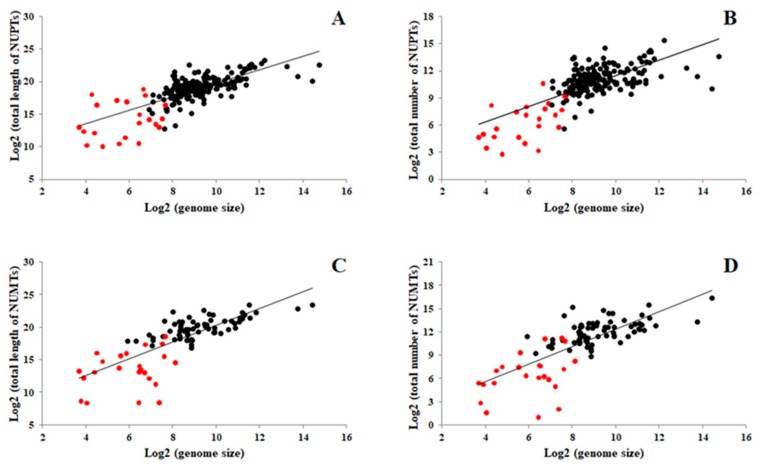
Correlation analysis between nuclear genome size and nuclear integrants of plastid DNA (NUPT)/nuclear integrants of mitochondrial DNA (NUMT) content or number in plants: (**A**) genome size versus cumulative length of NUPTs; (**B**) genome size versus total number of NUPTs; (**C**) genome size versus cumulative length of NUMTs; (**D**) genome size versus total number of NUMTs. This dot-plot was generated based on the data presented in Tables S1 and S2. The red dots indicate the results of algal plant analysis, whereas the black dots represent those of analysis involving other plants, most of which are flowering plants.

Different studies on the correlation between NUPT/NUMT abundance and nuclear genome size show conflicting findings [29,37]. Thus, correlation analysis between genome size and cumulative length or total number of NUPTs and NUMTs in more than 200 plant species was conducted to investigate whether nuclear genome size affect NUPT/NUMT content. A positive correlation existed between nuclear genome size and cumulative lengths of NUPTs/NUMTs, as well as the total number of NUPTs/NUMTs (Figure 1). Previous searches detected no such correlations, probably because of the smaller number of plant nuclear genomes analyzed. No correlations were detected between NUPT/NUMT content and chloroplast/mitochondrial genome size (Tables S1 and S2).

### 2.2. Organization and Distribution Patterns

NUPTs and NUMTs are frequently organized as clusters [21,38,39]. For example, we observed that approximately 45% of the 3155 NUPT insertions were organized in clusters in the genome of *Asparagus officinalis* [21]. In the model plant species *Arabidopsis* and rice, NUPTs and NUMTs are frequently nonrandomly arranged as loose clusters or tight clusters based on the physically linked degrees [39]. NUPTs/NUMTs are organized into three major patterns in plants: (a) continuous fragments of nuclear DNA collinear with mitochondrial or chloroplast DNA, (b) rearranged NUPTs/NUMTs originating from different regions of one organelle genome with non-uniform orientation, and (c) mosaics containing both NUPTs and NUMTs [40]. The presence of mosaic clusters containing NUPTs and NUMTs indicates that DNA fragments from different organelles might have concatemerized before insertion, or these nuclear regions are hotspots for integration [39,41]. Other organization patterns such as NUPTs/NUMTs with tandem duplications originating from one organelle fragment with the same orientation were occasionally observed [21]. These various organization patterns of NUPTs/NUMTs existing in plant genomes suggested that the origins and the evolutionary paths of the integrated regions may be different, and NUPTs/NUMTs are involved in shaping the plant genome via complicated mechanisms.

NUPTs/NUMTs are usually distributed unevenly in the analyzed plant genomes [20,21,39]. NUPTs and NUMTs are more preferred to distribute in centromeric and pericentromeric regions [36,39], which have few genes and a high level of heterochromatin content [42,43,44]. Such regions may offer a stable genomic environment for the maintenance of the alien organelle-originated DNA [16,36]. The integrations in these regions should be less harmful than those in other chromosomal regions [36]. For example, in rice and *A. officinalis*, large NUPTs are predominately distributed in the pericentromeric regions of the chromosomes [21,36]. In some species, such as *Arabidopsis* and sorghum, a considerable fraction of NUPTs and NUMTs is co-localized with transposable elements (TEs) [16]. These findings imply that recombination based on repetitive sequence can lead to the rearrangement of chromosome structure and contribute to the various organization patterns of organelle-derived sequences.

The chromatin state seems to be an essential factor that affects the successful insertion of organellar DNA into nucleus. The pre-insertion status of *Oryza sativa* subsp. *indica*-specific NUPTs suggests that the newly transferred organellar sequences are predominantly inserted into open chromatin. This phenomenon has also been observed in humans [45]. However, current existing NUPTs/NUMTs are often detected in heterochromatin regions. Such paradox can be explained by two reasons. One is that the accessibility of chromatin can be modified by external environment, such as stress [46], and/or by genetic crash, such as hybridization [47]. Alternatively, many new insertions in the open chromatin may not be retained because of selective pressure; for that, the insertion into exons of genes can damage gene function. Indeed, most NUPTs/NUMTs are located in introns or untranslated regions [23]. By contrast, the heterochromatin regions are more facilitated for the maintenance of organelle-derived sequences.

### 2.3. Modification Pattern

As alien gene materials of the nuclear genome, NUPTs/NUMTs can cause host genome instability; they may lead to genomic region reshuffling, genome size expansion, and heterochromatization [16,38,48]. Epigenetic regulation, mainly including DNA methylation and histone tail modifications, provides a defense measure for inhibiting the activity of mobile DNA and other types of extraneous DNA [49,50].

The mutation patterns of NUPTs of a number of plant species show biased mutations of cytosine (C) → thymime (T) on one DNA strand, and guanine (G) → adenine (A) substitutions on the opposite strand [4,33,38,51]. These biased substitutions might be due to the hypermethylation of cytosine residues with subsequent deamination [52]. These observations suggest that DNA methylation might play essential roles in regulating integrated organellar DNAs, mainly associated with the transcription suppression of integrated organelle DNA [53,54]. In fact, it has been revealed that a considerable number of NUPTs are methylated, and the DNA methylation intensity and level decrease over evolutionary time. The DNA methylation-modified NUPTs may maintain the stability of plant nuclear genomes against the insertion of organellar DNA sequences and play an important role in the symbiosis of nuclear and organelle genomes [51]. Further methylome data analysis of epigenetic mutants in *Arabidopsis* and rice show that organellar DNA sequences are methylated mainly via the maintenance methylation machinery, involving *DDM1*, *CMT3*, *CMT2*, and *SUVH4*/*KYP*. However, other mechanisms, such as RNA-directed DNA methylation and homology-dependent DNA methylation machinery may also play roles in the methylation level.

### 2.4. Transfer Rate of Organellar DNA to Nucleus

The frequency of organellar DNA transfer is positively correlated with the number of mitochondria or plastids in the cell [55,56,57]. Two independent laboratories established a similar screen experimental system to measure the rate of nuclear DNA transferred from plastids; this system was based on transplastomic lines containing a selectable marker gene, whose transfer to the nuclear can confer antibiotic resistance of the regenerated or outcrossed progenies [10,12]. De novo NUPT formation occurred once in approximately 16,000 pollen grains [10] or once in every five million somatic cells screened [12]; this result suggests the high frequency of DNA transfer from the chloroplast to the nucleus. Such high frequency of organellar DNA transfer to the nucleus was also observed in yeast, in which the transfer rate of the mitochondrial DNA to the nucleus is approximately 2 × 10^−5^ per cell per generation [58]. Abiotic stresses, such as subtle heat or cold treatment, can even increase the transfer frequency [55,59]. It is noteworthy that in these experiments, the estimated frequency of DNA transfer from the organelle to the nucleus may be fewer than in reality, because the screen system only focused on the transfer of the selectable marker gene from the organelle genome; those events where the transferred organellar genome fragment did not contain the selective gene may have been missed.

The frequency of plastid-to-nucleus DNA transfer differs markedly among diverse tissues [10,12,60]. The transfer frequency in gametophytic tissue is higher than that in somatic cells. Specifically, the transfer frequency is much higher in male germlines (1 per 11,000 pollen grains) than that in female germlines (1 stable transposition in 273,000 ovules) [60]. The elevated frequency examined within male germline may be caused by the fact that the chloroplast DNA within pollens are more likely to degrade, which was examined in a number of plant species [61].

## 3. Genetic Consequence and Fate of NUPTs/NUMTs in Plants

**Figure 2:**
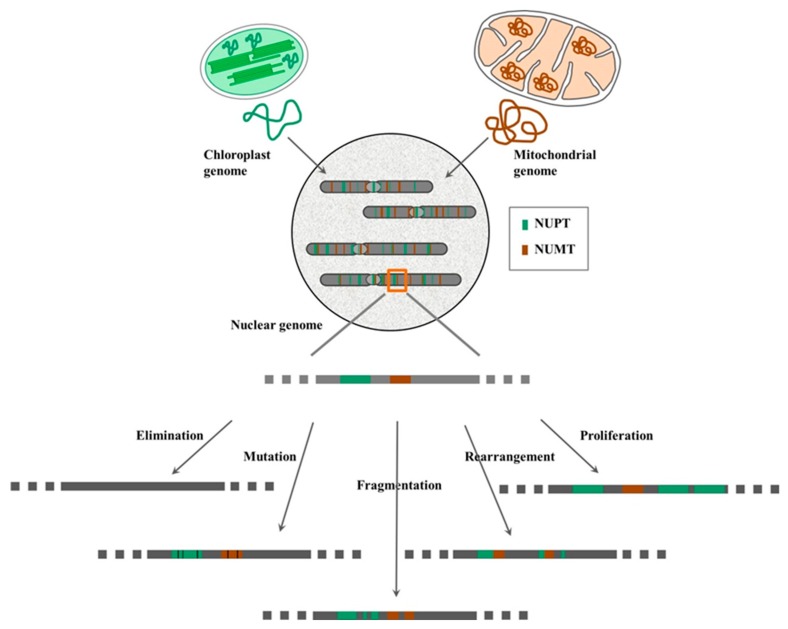
Diagrammatic sketch summarizing the fate of integrated organellar DNA in nuclear genome. See details in text.

Once the organellar DNA has integrated into the nuclear genome, the inserted sequences are placed into a new environment and will undergo gradual amelioration to adapt to the host genome. Most of the NUPTs/NUMTs begin to decay because they are not affected by selection pressure [33,38,40]. Analysis of the organization, dynamics, and interspecies variation of current NUPTs/NUMTs in plant genomes suggests the following five post-integration fates of organellar DNA: (a) elimination, (b) mutation, (c) fragmentation, (d) rearrangement, and (e) proliferation (Figure 2).

### 3.1. Elimination

Considering the high transfer frequencies of organellar DNA to nuclear genome and the relatively low percentage of NUPTs/NUMTs in the plant genome, it is expected that a considerable number of these transferred organellar DNA fragments would be eliminated to prevent rapid genomic enlargement. The fate of the newly integrated chloroplast fragments (kanamycin resistance gene, *neo*) in tobacco reveals that four of nine lines show different level of kanamycin resistance instability, which is caused by the deletion of *neo*. The loss of the organellar transferred fragments usually occurs during mitosis, because intraspecific variation of NUPTs is observed frequently. However, it can also occur during meiosis occasionally [48,62]. A comprehensive analysis of the NUPTs existing in the rice genome showed that 80% of the NUPTs were eliminated within a million years following their integration. The average half-lives of these NUPTs are estimated to be 0.5 million years (Myr) for large insertions and 2.2 Myr for small insertions. Thus, the nuclear genome is in balance between continual integration and fast deletion of the organellar DNA [36]. The rapid elimination is a counterbalance mechanism of the frequent integration of plastid DNAs. Investigation of organellar DNA integrants in the nuclear genomes of *Arabidopsis* and rice have suggested that replication slippage is a mechanism for such deletion events, because short direct repeats are found frequently in the flanking regions of the deleted sequences [33,38].

### 3.2. Mutation

As an intrinsic property of DNA, mutation is a predominant post-integration fate of organelle DNA. Organelle-derived sequences in the nuclear genome are unavoidably subject to the evolutionary forces that act on nuclear DNA [4]. Given that mutation rates vary greatly among organellar and nuclear DNAs, the integrated sequences show different mutation rates compared with the organellar sequences that they derive. In most animals, the mutation rate of mitochondrial DNA is approximately ten times faster than that of the nuclear DNA [63,64,65]. Thus, NUMTs inserted into the nucleus reserve ancestral mitochondria information and become “molecular fossils” that can be useful phylogenetic markers to study the evolutionary route of related taxa [66,67]. By contrast, the mutation rate of organellar DNA in plant species is much slower than that in the nuclear genome [68]. Organellar DNA is evolved with approximately one or fewer mutations per kb per million years [69], and the mutation rate is approximately seven mutations per kb per million years for the nuclear genome [70]. Thus, the NUPTs/NUMTs inserted into the plant nuclear genome evolve much more rapidly. With the evolutionary process, NUPT/NUMT mutation accumulates until they are not recognized as NUPTs/NUMTs because of the large sequence divergence between the evolved integrated sequences and the ancestral organellar DNAs. A previous investigation of recently acquired insertions of nuclear organellar DNA in *Arabidopsis* and rice revealed that the C → T and G → A transition mutations have occurred far more frequently than other point mutations. The 5-methylcytosine hypermutation frequency is approximately 5.6-fold and 9.5-fold higher than other point mutations in *Arabidopsis* NUMTs and rice NUPTs, respectively. In addition, most of the short insertion and deletion mutations occurred at homopolymeric regions and were less frequent than point mutations [33,38]. Thus, the 5-methylcytosine hypermutations may be a major mechanism of mutational decay for the newly integrated organellar sequences.

### 3.3. Fragmentation

As discussed above, the sequence similarity between NUPTs/NUMTs and their original chloroplast/mitochondrial DNAs will decrease over evolutionary time. Such sequence similarity is correlated positively with the size of NUPTs/NUMTs. In addition, dot plot analysis showed many separate but tightly linked NUPTs, which are collinear with the chloroplast genome in *A. officinalis* [21]. These pieces of evidence indicate that the original insertions are large and then break into smaller fragments [36,39]. The insertion of TEs and other non-organelle DNA sequences into NUPTs and NUMTs plays a significant role in this fragmentation process [36,39]. Some NUPT/NUMT clusters were formed by such process of organellar DNA insertion and subsequent fragmentation accompanied by incorporation of non-organellar DNA.

### 3.4. Rearrangement

A considerable number of NUPTs/NUMTs are arranged in tandem arrays of sequences originating from various regions of the plastid or/and mitochondrion genome (s); this phenomenon suggests that the transfer of organellar sequences undergo rearrangement before and/or after their insertion into the nuclear genome. In rice, the fragmentation and reshuffling events could occur immediately after plastid DNAs are integrated into the nuclear genome [36]. A detailed analysis of the *A. thaliana* 620-kb large NUMT shows that at least four rearrangement events have occurred, among which, three were localized at the initial 76 kb of the insertion [36]. These rearrangements may have occurred after integration, but they can also occur before transfer in the organelle via homologous recombination between repetitive sequences [71] or in the nucleus either before or during integration [11,39,72,73].

### 3.5. Proliferation

The integrated organellar DNAs can amplify themselves to increase the copy number in the nuclear genome. The amplified fragment can be arranged in tandem arrays or distributed separately in the host genome. For example, in *A. officinalis*, we identified several NUPT clusters arranged by a number of nearly identical chloroplast DNA sequences [21]. In *Carica papaya*, a plastid-derived sequence containing *rsp15* gene has been proliferated 23 times in the hermaphrodite-specific regions of the Y^h^ chromosome (HSY) [74]. The NUPTs/NUMTs themselves are not able to duplicate; thus, their proliferation in the nuclear genome is probably duplicated, co-amplified with neighboring retrotransposons, which can easily increase their copy number via “copy-and-paste” retrotransposition. Indeed, intact *Ty3-pypsy* retrotransposable elements are presented less than 1.5 kb upstream of most *rsp15* NUPT sequences, suggesting that the NUPT proliferation is probably mediated via retrotransposons [74].

We summarized the various fates of the organellar DNA integrated into the host nuclear genome. It is worth noting that the occurrence of the abovementioned events is not isolated and that these events usually occur integrated within one NUPT or NUMT sequence. Thus, after the organellar DNA integrated into the nuclear genome, they are fragmented, shuffled, and accompanied by nucleotide mutation. During this process, a considerable number of NUPTs/NUMTs are deleted from the nuclear genome. Thus, the current repertoire of NUPTs/NUMTs is in rapid variation of fragmentation, mutation, and rearrangement and in dynamic equilibrium between continuous integration and frequent deletion.

In addition, we only discuss the fate of the NUPTs/NUMTs with simple origin, that is, individual organellar fragment from chloroplast or mitochondrial genome transferred to the nucleus. However, the transferred sequence not only comes from one fragment of plastid or mitochondria but it can be derived from both plastids and mitochondria. In this case, the disparate fragments from plastids and mitochondria are combined and/or rearranged and then integrated into the nuclear genome [38,62]. Such integrants can undergo the abovementioned various evolutionary events and complicate the structure of NUPTs/NUMTs.

## 4. Effects of Organelle DNA-Derived Sequences on the Nuclear Genome Structure and Evolution

Organellar DNA integration provides novel source of plant genome evolution. In general, NUMT/NUPT insertions seem to expand the genome size due to the incorporation of novel DNA, although some of the newly integrated sequences can be lost through deletion. A recent study showed that the incorporated plastid DNA can be deleted and accompanied by the loss of adjacent non-plastid-derived DNA within one generation of transfer [62]. Thus, the integration of organellar DNA and its subsequent diverse variation events could contribute significantly to the dynamic nuclear genome evolution.

### 4.1. Contribution to the Genetic Diversity

Organellar DNA integration is a substantial and ongoing process that generates genetic diversity. The NUMT/NUPT numbers, sizes, and densities show large divergence between different species. Even in the same species, different lines or ecotypes represent sequence variation of NUMTs and NUPTs. Among the 40 investigated NUMTs in humans, 12 are polymorphic [29]. In *Arabidopsis*, a 3.9-kb NUMT is presented in a polyubiquitin gene in four ecotypes, including Columbia, Eifel, Enkheim, and Hilversum [75,76]. However, this NUMT loci did not exist in five other ecotypes investigated [75]. Another 104-bp rearrangement NUMT derived from different mitochondrial DNA fragments is detected at the subtelomeric region of the short arm of chromosome 1 in 12 out of the 35 *Arabidopsis* accessions examined [77]. Similar results were found in rice and maize. A NUPT with length of 131 kb was detected on chromosome 10 of *O. sativa* subsp. *japonica*, but not detected in *O. sativa* subsp. *indica* or *O. rufipogon* nuclear genomes [33]. Fluorescence in situ hybridization (FISH) analyses using overlapping mitochondrial/chloroplast DNA fragments as probes revealed that the number and positions of detectable NUMTs/NUPTs varied markedly among different maize inbred lines [13,78]. For example, a mix of cosmids containing overlapping mitochondrial DNA fragments was probed, and only B73 showed a strong hybridization signal on 9L among the examined inbred lines; thus, this site may represent a recent mitochondrial DNA insertion [78]. Some NUMT sites are diverse even within the same maize inbred line collected from different laboratories (sources) [78]. Such diversity may be attributed to different mechanisms, such as integration of a new fragment of mitochondrial DNA, proliferation of an existing NUMT fragment, or deletion and degradation of NUMT sequences at a particular site [78]. The interspecific diversity in NUPT and NUMT accumulation might be affected by the number and stability of plastid and mitochondria in the germline; another factor is the species-specific mechanisms regulating nuclear DNA acquisition and deletion [37]. These results strongly suggested that the organelle DNA transfer is a frequently ongoing process, and the insertions of organelle-derived segments are components of the dynamic fraction of plant nuclear genomes.

### 4.2. Effects on Gene Structure and Evolution

Certain NUPTs and NUMTs are related to genes. For example, two NUMTs are located within genes in the yeast genome [79]. In the *Arabidopsis* and rice genomes, approximately 25% of NUMTs and NUPTs are found within genes [39]. In some cases, NUMT/NUPT sequences that fuse to non-organelle-derived nuclear DNA are transcribed, suggesting that NUMTs/NUPTs can remodel genes and their products by providing novel exons [19,80,81]. The mosaic genes with one or more exons originated from organellar DNA were analyzed in detail in yeast, *Homo sapiens*, *Arabidopsis*, and rice. In that study, a total of 474 NUMTs and NUPTs were detected within, or adjacent to the annotated genes. Among these NUMTs and NUPTs, 45 insertions are involved in a total of 49 protein-coding exons belonged to 34 genes [23]. Moreover, considering that the sequence divergence between NUMT/NUPT and the donor organellar sequences was constantly enlarged with evolutionary time, some organelle-originated exon sequences may be difficult to directly detect through conventional methods. Thus, organelle-derived DNA integrations might contribute to many ancient functional exon acquisitions that are not found thus far [23]. In addition, the Ka/Ks ratios (non-synonymous substitutions/synonymous substitutions) of the nuclear protein-coding exon derived from organellar reading frames are higher than 1, thereby suggesting the non-neutral evolution of these integrants and their adaptation to their novel functions [23].

In addition to recruiting as exons, NUMTs/NUPTs can also be associated with the regulatory elements of genes. Some organellar DNA sequences are integrated into introns, as well as regions of 5′ and 3′ to nuclear genes; these integrated sequences might be associated with changes in the gene regulation [80]. Specifically, two organelle-derived sequences, namely, *Arabidopsis* enhancer 12-7 and 12-Q, make the reporter genes show cell-specific expression in transgenic tobacco [82]. However, the influence of the organellar insertions on gene expression still needs further investigation.

### 4.3. Roles Played in the Sex Chromosome Evolution

Several reports revealed that NUPTs and NUMTs are strongly accumulated in the sex chromosomes of dioecious plants than other plants [74,83,84]. The first discovery of this phenomenon is in the model dioecious plant *Silene latifolia*, in which a bacterial artificial chromosome clone containing partial plastid genome sequences shows strong hybridization signals on the Y chromosome; however, the signals are very weak on the X chromosome and the autosomes. The large size of the Y chromosome in this species may be partially due to the accumulation of such NUPTs [83]. In another dioecious plant, *Carica papaya*, NUPTs are localized within the male-specific region of the Y chromosome (MSY) and HSY approximately 12 times the rate in the X chromosome counterpart and four times that of the genome wide average [74]. NUPTs/NUMTs accumulate in the sex chromosomes of other dioecious plants, such as *Rumex acetosa* [84] and *Coccinia grandis* [85]. In *A. officinalis*, the number and total length of NUPTs in Y chromosome were larger than those in other chromosomes. Sequence alignment and cytogenetic analysis show a 47-kb centromeric region in the Y chromosome with very high density of NUPTs, i.e., more than three-quarters of the sequences of this region originate from the plastid. However, the MSY and the nearby regions did not have NUPT integrations. Thus, NUPTs may play important roles in the centromere building of the sex chromosome of *A. officinalis* but are not implicated in MSY formation [21]. These studies reveal that the organellar DNA accumulation is strongly related to the evolution of plant sex chromosomes.

These integrated organellar DNA sequences are predicted as one of the evolutionary forces that drive the formation of sex chromosomes in dioecious plants. It is believed that the sex chromosomes originate from autosomes, and the evolutionary process involves some essential events, such as sex-determining gene emergence, recombination restriction, and Y chromosome degeneration [86]. In addition, sex chromosomes can recruit abundant TEs and organellar DNAs [74,85], and these novel sequences may benefit the structural differentiation and recombination restriction of sex chromosomes and contribute to Y chromosome degeneration [49]. The gathering of such novel sequences in sex chromosomes can promote recombination suppression of the sex chromosomes; when recombination is suppressed, such sequences can accumulate rapidly in the recombination-free region. The lack of recombination and release from purifying selection avoids their deletion from the recombination-suppressed region [87,88].

## 5. Mechanisms of Organellar DNA Integration

Organellar DNA sequences can be transferred to the nucleus through two distinct pathways, namely, direct DNA integration [89] and RNA-mediated DNA transfer via reverse transcription. Some experimental and bioinformatics studies suggest that many NUPTs/NUMTs are very large and include intergenic and/or non-coding regions of organellar DNA [11,37,38,89,90]. Thus, the migration of organellar DNA fragments into the nucleus may be mediated by DNA. However, the functional organellar gene relocated to the nuclear genome with splicing and RNA editing, and this event seems to have occurred before transfer; this phenomenon suggests the involvement of RNA intermediates [91,92,93]. Thus, the two pathways may both have important roles to play in the transfer process.

DNA escapes from organelles and its incorporation into the nucleus has been experimentally investigated in yeast [58,79] and tobacco [10,12]. Bioinformatics analysis and direct experimental evidence suggest that nonhomologous end-joining mechanism (NHEJ) is involved in organellar DNA incorporation during double-stranded break (DSB) repair. In yeast, fragments of mitochondrial DNA segments are captured into DSBs in the nuclear genome during the DSB healing process via NHEJ [79,94]. DSBs can be induced in vivo by exogenous and endogenous factors; they are the most potentially deleterious type of DNA damage [95,96,97]. DSBs can be usually repaired via the insertion of filler DNA into the lesion, whereas organellar DNA can be passively used as the filler DNA. The integration includes two DSB repair events, which are mediated by the short sequence microhomology (1–7 bp, “micro-identities”) between the break chromosome ends and organellar DNA or by a blunt-end repair without homology [79,94,96,98]. A similar mechanism involving NHEJ at DSB sites was examined in tobacco [45,55,62]. The integrated fragments are usually complicated mixtures of multiple different segments of chloroplast DNA end joined together based on sequence microhomology [62]. These results suggested that linking of linear DNA fragments generates chimeric DNA molecules before or during insertion into the nuclear genome [38,62]. Sequence analysis revealed that such terminal micro-identities have also been presented for NUMT integrants in humans [99,100], indicating that organellar fragments integrating into the nuclear genome via NHEJ during DSB repair process might be a common mechanism conserved in eukaryotic organisms. A recent study has shown that several mosaic organellar integrants present signatures of long homology; thus, other mechanisms, such as homologous recombination may also contribute to organellar DNA integration [40].

## 6. Conclusions and Perspective

Organellar DNA integrated into the nuclear genome plays important functional roles in gene and genome evolution. The characterization and distribution patterns of organellar-derived sequences of the nuclear genome are crucial to the elucidation of the dynamics and evolution of the genome in plants and other eukaryotes. Nevertheless, many unanswered questions about organellar integrated nuclear sequences remain. For example, the precise mechanism of organellar DNA transfer, the extent and variety of the effects of organellar DNA transfer on gene activity regulation, and the genome instability and defense mechanism caused by organellar DNA transfer are still poorly understood. Thus, bioinformatics approaches, cytogenetic analysis, and experimental studies illustrating transfer events are necessary to answer these questions.

Currently, the experimental analysis of DNA transfer from organelle to nucleus has been performed only in tobacco and yeast. Studies that used these model organisms have presented considerable information on NUMT/NUPT insertion events, such as the transfer frequency, the size of transferred genome fragments, the environmental conditions favoring the transfer, and the mechanism of transfer. However, that such events are universal is not yet certain and should be further studied in other plant species. Experimental models involving other plant species should be established to provide more insights into the transfer event.

Similar to TE, organellar DNA integrated into nuclear genome is considered alien genetic material to the nuclear genome. Such sequences could result in nuclear genome instability, and the nuclear genome can initiate defense mechanisms [49,50]. A recent report showed that DNA methylation might play an important role in this process [51]. Future studies on DNA methylation and heterochromatization of organellar DNA insertions may improve our understanding of the organellar DNA integrants involved in dynamic genome evolution.

## Supplementary Materials

The following are available online at https://www.mdpi.com/1422-0067/21/3/707/s1, Table S1, information of genome size, chloroplast genome size, and NUPT statistics results in sequenced plants. Table S2, information of genome size, mitochondrial genome size, and NUMT statistics results in sequenced plants.
