# Supplementary material for: Nuclear Integrants of Organellar DNA Contribute to Genome Structure and Evolution in Plants
Source: Int J Mol Sci. 2020 Jan 21;21(3):707. doi: 10.3390/ijms21030707 (PMC7037861; doi:10.3390/ijms21030707)
Supplement: Supplementary file 1 [file ijms-21-00707-s001.zip › proofed-ijms-692979-supplementary/Supplementary materials/Supplemental file 1.docx]

**Methods**

Detection of NUPTs and NUMTs

NUPT and NUMT insertions were detected using the BLASTN local alignment tools in the BLAST program package (version 2.2.31) with chloroplast or mitochondrial genome sequences as the query and nuclear genome data as the database. The parameters were as follows: -dust no, e-value threshold of 1e-4, mismatch penalty of –2, and word size of 9. The BLAST hits for the NUPTs that originated from the inverted repeat (IR) region of the chloroplast genome were counted only once because BLAST hits were obtained in both IR regions and could not be distinguished.
